# Post-operative Stereotactic Radiosurgery of Malignant Melanotic Schwannoma

**DOI:** 10.7759/cureus.22849

**Published:** 2022-03-04

**Authors:** Jennifer C Hall, Steven D. Chang, Thomas J Wilson, Kristen N Ganjoo, Angus Toland, Hannes Vogel, Erqi L Pollom, Elham Rahimy

**Affiliations:** 1 Department of Radiation Oncology, Stanford University, Stanford, USA; 2 Department of Neurosurgery, Stanford University, Stanford, USA; 3 Department of Medical Oncology, Stanford University, Stanford, USA; 4 Department of Clinical Pathology, Stanford University, Stanford, USA

**Keywords:** adjuvant therapy, radiation, radiotherapy, stereotactic radiosurgery, peripheral nerve sheath tumor, melanotic schwannoma

## Abstract

Melanotic schwannoma is an extremely rare schwannoma variant with malignant potential, demonstrating high local and distant recurrence. Given the paucity of data, recommended treatment with localized disease is radical resection, with the unclear benefit of adjuvant therapy. We present a case of an 18-year-old female with no past medical history or genetic syndromes who underwent margin-positive resection of an S1 nerve root melanotic schwannoma followed by adjuvant stereotactic radiosurgery (SRS). SRS was delivered without acute or late toxicity by 2.5 years post-treatment. She remains without evidence of recurrent disease, although longer follow-up is needed given the risk of late recurrence. Our case adds to the limited literature documenting the efficacy of adjuvant radiotherapy in melanotic schwannoma and is the first to describe the successful use of SRS for this rare disease.

## Introduction

Melanotic schwannoma is a rare variant of schwannoma with less than 200 reported cases, often presented in case reports or series [1-5]. It tends to occur in spinal and cranial nerves and comprises <1% of primary peripheral nerve sheath tumors [6]. It was historically thought to be benign and indolent, but more recent investigations have shown malignant potential, with local and distant recurrences as high as approximately 40% each [1,7]. Even in the absence of malignant histologic features, tumors may demonstrate aggressive clinical behavior [7]. Hence, it has been proposed to rename these tumors as malignant melanotic schwannomas (MMS) [1].

Given the rarity of this tumor, there is a paucity of data to guide optimal treatment. For localized disease, radical resection is preferred as incomplete resection is associated with tumor recurrence [6,8]. However, given these tumors often involve the nerve roots and are infiltrative, acquiring negative margins can be difficult without causing significant morbidity [8]. Radiotherapy and systemic therapy can be considered in these cases; however, robust data demonstrating efficacy are lacking given the rarity of this disease [1,5,7-9].

Given the unpredictable biologic behavior and initial reports of benign behavior, unclear efficacy of radiotherapy, and typically young age of these patients (median age in the fourth decade) [10], there are concerns with using adjuvant external beam radiotherapy. Stereotactic radiosurgery (SRS), which uses ablative dosing with rapid dose fall-off to spare adjacent normal tissues, is commonly used for classic schwannomas [11]. Compared to conventionally fractionated external beam radiotherapy, SRS may limit toxicity, including secondary malignancies [12]. SRS has not been described in the treatment of MMS.

We describe a case of an 18-year-old female with a symptomatic MMS with microscopically positive margins after re-resection. She successfully underwent adjuvant SRS with no evidence of disease or radiation sequelae at 2.5 years post-treatment.

## Case presentation

**Figure 1:**
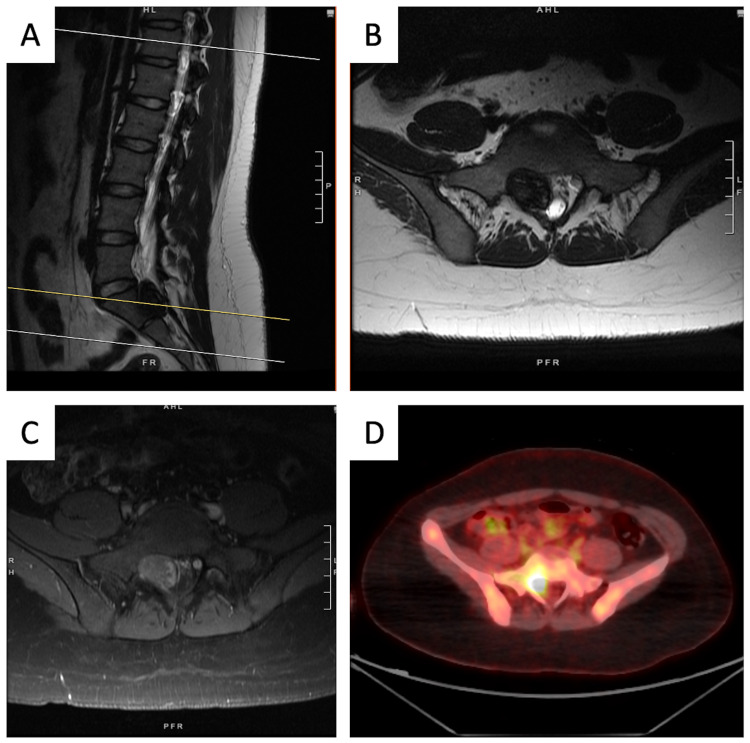
Preoperative imaging of right S1 malignant melanotic schwannoma. (A) T2 MRI sagittal showing T2 hypointense well-circumscribed lesion at the level of S1; yellow horizontal line demonstrates cross-cut for image (B) T2 MRI axial. The tumor appears primarily extradural and extends into the neuroforamen. The lesion is also (C) T1 hyperintense on MRI and (D) highly avid on positron emission tomography/computed tomography (PET/CT).

**Figure 2:**
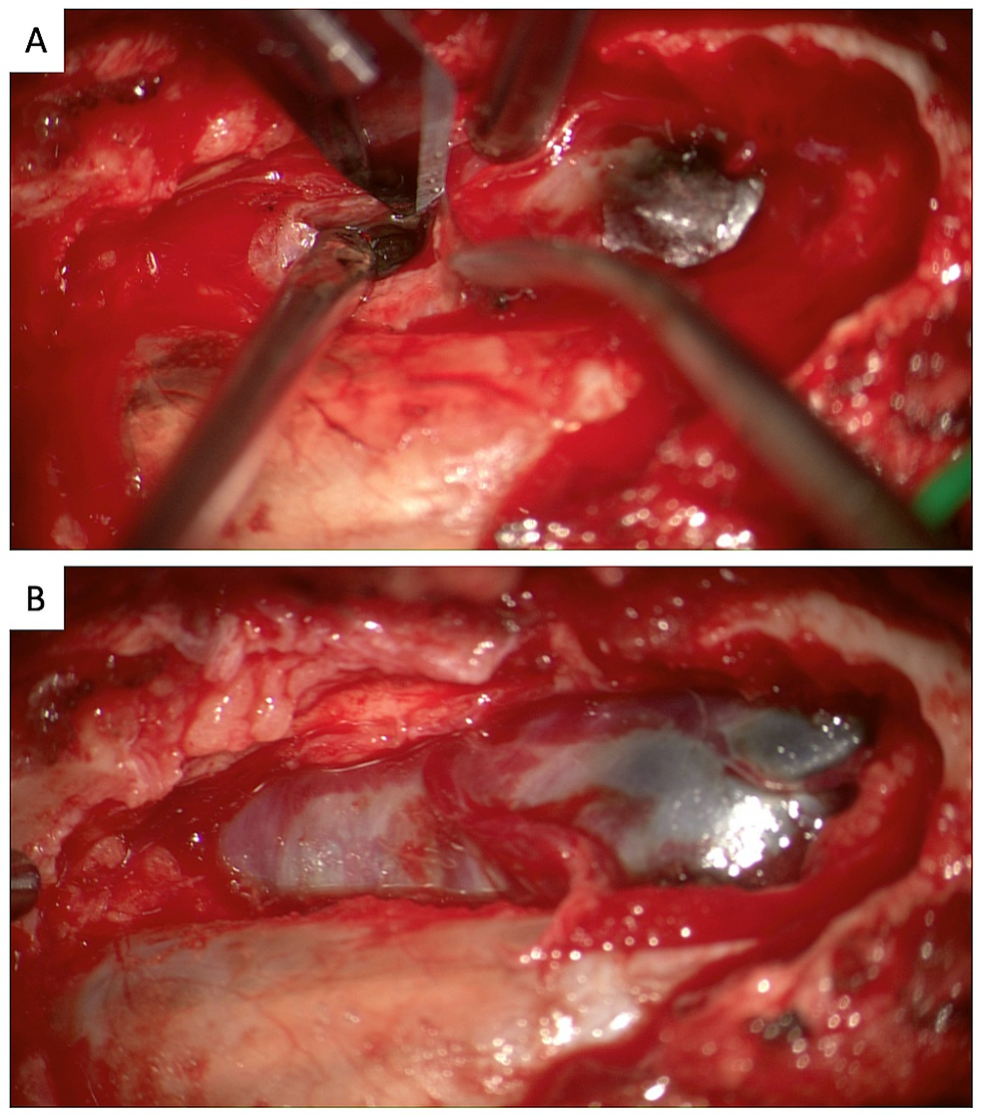
Intraoperative images from initial L5-S1 laminectomy with subtotal tumor resection. During the course of the operation, the ligamentum flavum was completely removed between a partial portion of L5 and sacrum. (A) At this point, the tumor was evident on the right. (B) After completion of S1 laminectomy, the tumor presents more clearly in view, black in color, and well encapsulated.

An 18-year-old female without significant past medical or family history presented with progressive lower back pain and right lower extremity radicular pain for several years. Magnetic resonance imaging (MRI) of the lumbar spine revealed a T1 hyperintense/T2 hypointense well-circumscribed 2.9 x 2.8 cm mass along the course of the exiting right S1 nerve root; it appeared to be extradural with extension into the S1-S2 neuroforamen and expansive remodeling of the neuroforamen and S1 vertebral body (Figures 1A-1C). On positron emission tomography/computed tomography (PET/CT), the lesion was fluorodeoxyglucose (FDG) avid (maximum standardized uptake value of 9), concerning for malignancy (Figure 1D). No distant lesions were identified. Initial percutaneous fine-needle aspiration was inconclusive. Subsequent L5-S1 laminectomy with subtotal resection confirmed malignant melanotic schwannoma (Figure 2).

The recommendation upon multidisciplinary review was aggressive re-resection, including the sacrifice of the S1 nerve root. The S1 nerve root was ligated at the dural margin and distal to the mass to allow en bloc, gross total resection of the mass. The proximal margin was microscopically positive for the tumor. Genetic testing was done, primarily to rule out Li-Fraumeni syndrome and Carney complex. Stanford solid tumor actionable mutation panel (STAMP) did not identify mutational targets.

Adjuvant radiotherapy was recommended to minimize the risk of local recurrence. To minimize the risk of secondary malignancy, infertility, and overall treatment burden in this young patient, adjuvant SRS was recommended over external beam radiotherapy. Systemic therapy was not recommended given localized disease and unclear benefits.

Stereotactic radiosurgery treatment

**Figure 3:**
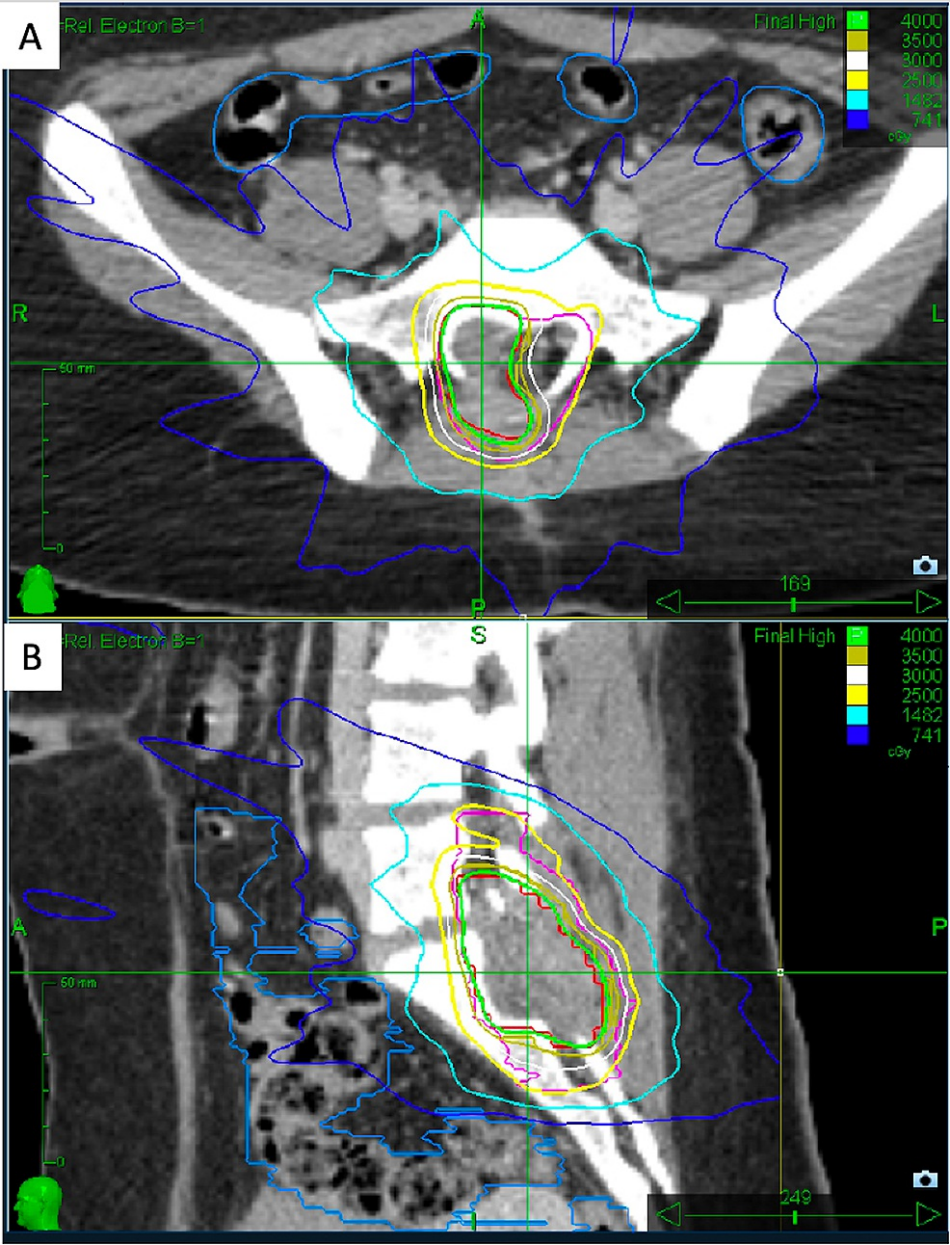
Stereotactic radiosurgery plan. (A) Axial cut through clinical target volume (CTV) 40 Gy (red) and CTV 25 Gy (pink) with colored isodose lines (100% isodose line is light green). (B) Sagittal view.

Radiation simulation was performed three months post-operatively, after restaging MRI of the full neuroaxis confirmed no interval distant metastases. The resection site demonstrated post-operative changes and likely granulation tissue. The pre-operative T2 MRI was fused for target contouring. A clinical target volume (CTV) 40 Gy was contoured as the post-operative bed and ensured coverage of pre-operative tumor extent. A dose of 40 Gy in five consecutive daily fractions was prescribed to the CTV 40 Gy target periphery to the 84% isodose line. A subclinical CTV 25 Gy was also prescribed to cover the remainder of the spinal canal on axial sections, and approximately 1 cm craniocaudal margins on the visible post-operative changes (Figure 3).

**Figure 4:**
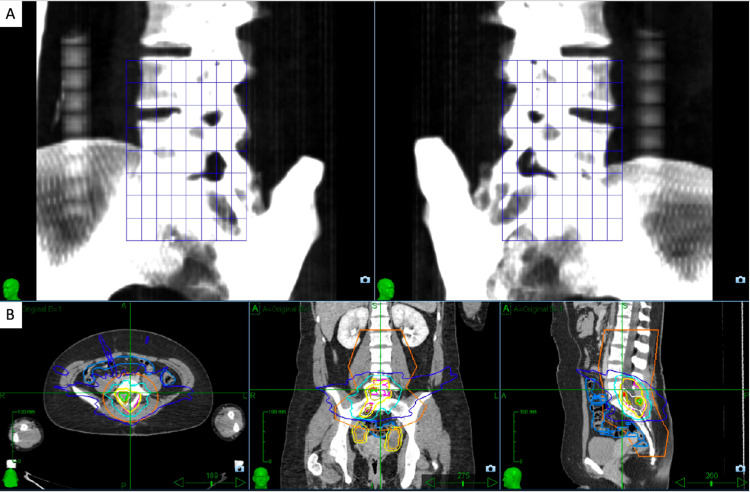
X-sight spine tracking of CyberKnife System for fiducial-less tracking. (A) Blue 8 x 8 grid of tracking nodes indicates appropriate matching of treatment kV and digitally reconstructed radiograph (DRR) library made from patient’s simulation CT scan. (B) The tracking region was defined as the lumbar and sacral spine (appearing within orange contour).

SRS was delivered on CyberKnife System (Accuray Incorporated, Sunnyvale, CA) with patient immobilization using a custom vacuum bag and real-time spine tracking (Figure 4), allowing for sub-millimeter accuracy [13]. Care was taken to minimize dose to the ovaries, with avoidance of beam path through the ovaries + 2 cm (to account for potential ovarian motion), and maximum dose to bowel minimized. The ovaries received a mean of 0.62 Gy while the bowel received a maximum point dose of 22.67 Gy. Given the large target size (CTV 40 Gy of 26 cc and CTV 25 Gy of 95 cc), and to minimize treatment time, multileaf collimators (MLCs) were used instead of circular collimators. The SRS plan achieved CTV coverage >95%, good conformity with an index of 1.14, and a treatment time of 22 minutes.

The patient tolerated radiotherapy without complication and did not require steroids following treatment. Three months after completion of radiosurgery, she reported significant improvement in pain and remained free from radiographic recurrence. She continued with regular surveillance every three months for the first two years, and every six months thereafter. At 2.5 years post-SRS, she is doing clinically well, with no weakness, resolution of her pain, and no evidence of disease on serial full-spine MRIs and PET/CTs.

## Discussion

Given the rarity of melanotic schwannoma, neither the biologic behavior nor the optimal treatment has been well classified. Discovered almost a century ago, it was initially thought to be indolent and benign. However, a recent series, the largest to date of 40 patients, demonstrated high local and distant recurrence of 35% and 44%, respectively [1]. Similarly, a recent literature review of 57 cases demonstrated local and distant recurrence rates of 15% and 26%, respectively. Most recurrences were within the first five years, although late relapses were reported [7].

**Figure 5:**
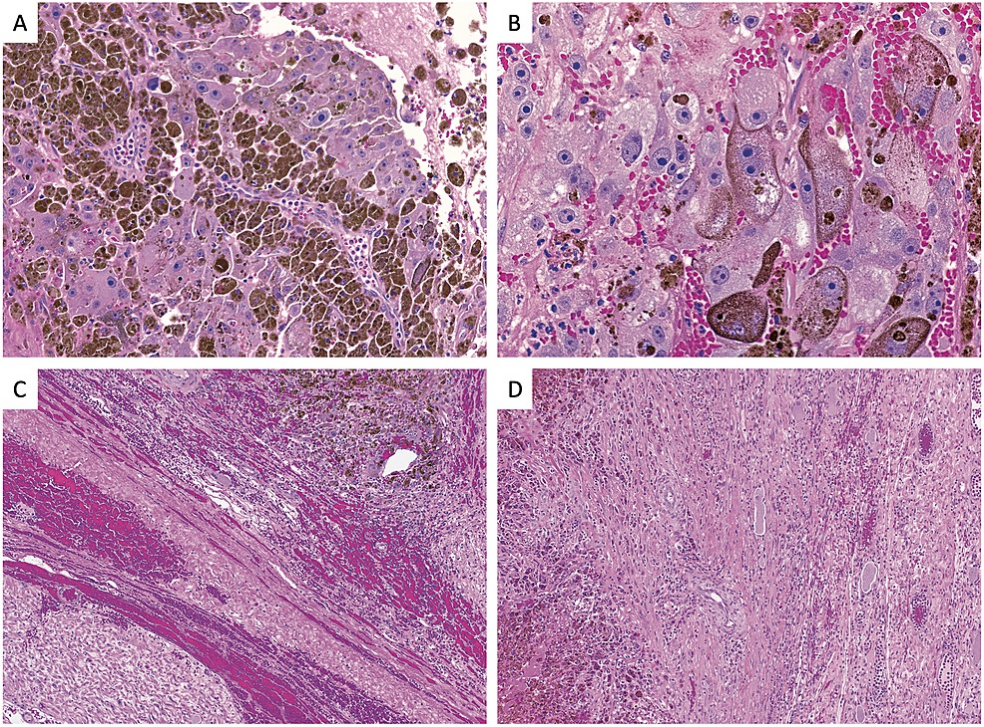
Histopathology demonstrating malignant melanotic schwannoma. Shown at original magnification (A) 20x and (B) 40x, neoplastic cells appear with a focal component of the oval to spindled morphology. Immunohistochemical stains reveal neoplastic cells are positive for HMB-45, S100, and Melan-A. A Ki67 stain highlights a low proliferation index (<5%). The specimen is shown at original magnification (10x) to demonstrate neoplastic cells arising in a background of uninvolved (C) ganglion cells and (D) adjacent nerve tissue.

There are two critical aspects of this case that should be emphasized. First is the assurance of accurate diagnosis and staging. Notably, melanotic schwannomas appear radiologically distinct from classic schwannomas and peripheral nerve sheath tumors given the T1 hyperintensity and T2 hypointensity on MRI (Figure 1), secondary to paramagnetic free radicals in melanin. Pathologic review and clinical assessment should be carefully done to distinguish melanotic schwannoma from malignant melanoma, which is immunophenotypically indistinguishable (both demonstrate positive staining for melanoma markers of HMB-45, S100, and Melan-A) [2]. In this case, intimate association with the nerve root, spindled morphology, low proliferation index (<5%, although melanotic schwannoma can demonstrate higher indices like melanoma) [2], and careful dermatologic examination (to ensure the absence of cutaneous melanoma) supported the diagnosis of melanotic schwannoma (Figure 5). With the diagnosis of melanotic schwannoma, staging is also critical given the unpredictable clinical behavior and concern for metastases. PET/CT may be particularly useful to distinguish those with high malignant potential, to rule out occult metastatic disease/recurrence, and to monitor treatment response [14].

The efficacy of adjuvant radiotherapy in melanotic schwannoma is unclear and limited to a few case reports of conventionally fractionated radiotherapy to as high as 60 Gy [2,4,6-8,15]. The role of radiotherapy after margin-positive surgical resection is controversial given the unclear efficacy, particularly given the discordance of early and recent reports on the malignant potential of melanotic schwannomas. In addition, as these patients are typically young [10], there is a concern for late toxicity of radiotherapy (including secondary malignancy, or infertility if radiating in the pelvis). Even if radiation is recommended, the optimal dose is unclear, with published reports ranging from 50 to 60 Gy over five to six weeks.

The second point of emphasis, to our knowledge, is that we describe the first use of SRS in the adjuvant treatment of this tumor. SRS was chosen to reduce treatment duration (from six weeks to one week) and to reduce irradiation of normal tissue due to the tight dose gradient. SRS allowed us to reduce the dose to the ovaries in this young, premenopausal patient, and to potentially reduce the risk of secondary malignancy [12]. The dose of 40 Gy in five fractions was chosen given the biological equivalence to 60 Gy conventionally fractionated, assuming tumor alpha/beta of 10. If melanotic schwannoma has a lower alpha/beta ratio, like that suggested for malignant melanoma [16], the biological equivalent dose of our SRS treatment may be even higher, further supporting the use of SRS over conventional fractionation.

Our patient is now 2.5 years out from SRS and is doing clinically well without radiation sequelae or evidence of disease. It should be noted that longer follow-up is required as late relapse after five years has been reported [7]. However, outcomes thus far are promising. This case adds to the very limited literature on radiotherapy use in this rare disease, and the first use of adjuvant SRS.

## Conclusions

Melanotic schwannoma is a rare schwannoma variant with a high potential for local and distant recurrence. The role of adjuvant therapy in cases where radical resection is not achieved remains unclear. This case highlights the potential efficacy of adjuvant radiotherapy in cases of marginal resection. More specifically, this case demonstrates adjuvant SRS can be an effective and well-tolerated treatment for resected melanotic schwannoma.
